# Analysis of Intestinal Bacterial Microbiota in Individuals with and without Chronic Low Back Pain

**DOI:** 10.3390/cimb46070435

**Published:** 2024-07-12

**Authors:** Antonio Martins Tieppo, Júlia Silva Tieppo, Luiz Antonio Rivetti

**Affiliations:** 1 Rehabilitation Service, School of Medical Sciences of Santa Casa de São Paulo, São Paulo 01221-020, Brazil; 2 Faculty of Medicine, University of São Paulo, São Paulo 01246-903, Brazil; 3 Postgraduate Cardiac Surgery Discipline, School of Medical Sciences of Santa Casa de São Paulo, São Paulo 01221-020, Brazil; luiz.rivetti@fcmsantacasasp.edu.br

**Keywords:** intestinal microbiome, intestinal microbiota, inflammation, chronic low back pain

## Abstract

Low back pain is a health problem that represents the greatest cause of years lived with disability. This research seeks to evaluate the bacterial composition of the intestinal microbiota of two similar groups: one with chronic low back pain (PG) and the control group (CG). Clinical data from 73 participants and bacterial genome sequencing data from stool samples were analyzed. There were 40 individuals in PG and 33 in CG, aged between 20 and 50 years and with a body mass index of up to 30 kg/m^2^. Thus, the intragroup alpha diversity and intergroup beta diversity were analyzed. The significant results (*p* < 0.05) showed greater species richness in PG compared to CG. Additionally, a greater abundance of the species *Clostridium difficile* in PG was found along with 52 species with significantly different average relative abundances between groups (adjusted *p* < 0.05), with 36 more abundant species in PG and 16 in CG. We are the first to unveil significant differences in the composition of the intestinal bacterial microbiota of individuals with chronic low back pain who are non-elderly, non-obese and without any other serious chronic diseases. It could be a reference for a possible intestinal bacterial microbiota signature in chronic low back pain.

## 1. Introduction

Low back pain (LBP) is a health problem that represents the greatest cause of years lived with disability [1]. It is estimated that 80% of the population suffers from low back pain at some point in their lives. It is the main factor limiting activity for people under 45 years of age and tends to increase in prevalence in younger people [2]. In many cases, its etiology, progression and chronicity cannot be fully explained [3].

The growing evidence of the presence of high levels of pro-inflammatory cytokines and pro-nociceptive neurotrophins in vertebral tissues (discs, facet joints and even cerebrospinal fluid) [4] may demonstrate the involvement of an overactive innate immune response in the genesis and progression of low back pain [5].

There has been an increase in preclinical and human studies on microbiota and the activity of its genes (microbiome), especially the intestinal microbiota. Correlations have been found in certain conditions such as dysbiosis [6], to the generation of chronic low-grade inflammation [7] associated with the pathogenesis of various diseases [8], including painful pathologies of the lumbar spine [9,10,11]. Yao et al. (2023), found through an experimental rat model of intervertebral disc degeneration (IDD) that the expression of tumor necrosis factor (TNF-α), interleukin (IL)-1β, IL-6, metalloproteinase (MMP)-3, MMP-13, nucleotide-binding oligomerization domain-like-receptor family pyrin domain-containing 3 (NLRP3) and caspase-1 increased in the rats of the IDD group. On the contrary, collagen II and aggrecan levels were downregulated. Additionally, vertebral disc tissue was severely damaged in the IDD group [9]. In their prospective literature review, Li et al. (2022), summarize three potential mechanisms by which the gut microbiota can induce IDD and cause LBP, such as (1) the translocation of bacteria across the gut epithelial barrier and into the IDD, (2) the regulation of the mucosal and systemic immune system, and (3) the regulation of nutrient absorption and metabolite formation at the gut epithelium and its diffusion into the IDD [10]. Rajasekaran et al. (2020) performed an experimental case-control study of genomic DNA from 24 lumbar intervertebral discs (IVDs). Their results showed eight normal, eight discs with herniation (DH) and eight discs with degeneration (DD) types, among all of which the authors found a rich bacterial presence. The varying biodiversity and abundance between healthy and diseased discs were documented, with protective bacteria being abundant in normal discs and putative pathogens abundant in DD and DH [11].

This research aimed to evaluate the bacterial composition of the intestinal microbiota of two groups, one of which is made up of people with chronic LBP, constituting the low back pain group (PG), and the other without, constituting the control group (CG). Moreover, we aim to determine whether there are relevant differences in the metrics of these bacteria. To accomplish these aims, the intra-group microbial alpha diversity and the inter-group beta diversity were assessed. Alpha diversity refers to the richness and abundance of bacterial species found in a sample; richness represents the total number of species. Abundance, in turn, refers to the proportion of each bacterial species in selected samples. Once the alpha diversity of each individual group has been obtained, it is possible to establish the beta diversity between the groups [12], that is, the difference between the total richness of taxa found and the difference in abundance of each species between CG and PG [12].

## 2. Materials and Methods

This paper presents an original observational case-control study carried out with people from the metropolitan region of the city of São Paulo, Brazil. There are no conflicts of interest to declare and the project was funded by the researcher.

### 2.1. Participants

This study was based on the collection of clinical data and the analysis of stool samples from 73 participants, subdivided into CG (made up of 33 asymptomatic individuals) and PG (made up of 40 individuals who have had chronic low back pain for at least 2 months). The groups were formed via analytical convenience sampling since anyone from the population of the metropolitan region of the city of São Paulo who met the eligibility criteria could be a participant, as well as due to the high financial cost of sequencing intestinal bacterial microbiota, and the need to focus on participants who were connected to the primary objective (assumption of relevance). The generated data then underwent statistical instrumentation. The selection of participants sought to achieve the greatest possible similarity between the groups in terms of gender, age, BMI and physical activity. All the volunteers included were adults aged between 20 and 50 years old with a body mass index of up to 30 kg/m^2^, among whom 47.4% and 46.7% had sedentary lifestyles in PG and CG, respectively. Pregnant women or those who had given birth in the last 3 months were not included. Participants with anatomical deformities and reduced spinal mobility detectable on physical examination were excluded. Those who had received antibiotics in the 3 months prior to the collection of the stool sample and who were continuously using non-hormonal anti-inflammatory drugs, glucocorticoids or antidepressants were excluded. People with acute infections, uncontrolled chronic diseases, diabetes, inflammatory bowel diseases or any other serious comorbidity were also excluded. Smokers and heavy drinkers (more than five doses of alcohol per week) were excluded.

### 2.2. Study Stages

The initial assessment of the participants included the following information: demographic data (age, gender, race, level of education, income); general and specific clinical examination of the spine; characterization of pain in the PG (temporality and application of the visual analog scale (VAS) of pain, graded from 0 to 10, in which 0 represented no pain, 1 very mild pain, 2 mild pain, 3 mild to moderate pain, 4 moderate pain, 5 moderate to severe pain, 6 severe pain, 7 severe to very severe pain, 8 very severe pain, 9 very severe pain to the worst possible pain and 10 the worst possible pain); pattern of nutrition and hydration; number of hours of sleep at night; physical activity; medication in use; route of delivery; and a self-completion of a form on intestinal habit characteristics (format using the Bristol stool scale [13], frequency of bowel movements and presence of symptoms such as oscillation between diarrhea and constipation). All the participants had their stool samples collected according to the Bioma4me* laboratory’s standards for stool metagenomic studies (https://bioma4me.com.br/genetica/processo-de-sequenciamento/, accessed on 1 November 2019). The genetic material contained in each individual sample was analyzed using the DNA amplification technique via PCR (polymerase chain reaction). A subsequent sequencing of the genes expressing the V3 and V4 hypervariable regions of the 16s portion of bacterial ribosomal RNA was conducted using RefSeq, which was made available by Illumina, Inc.’s Basespace (San Diego, CA, USA, https://www.illumina.com). This technique makes it possible to determine microbial composition at the species level. The reports generated provided the taxonomization of the bacterial microbiota through operational taxonomic units [14]. Based on the report issued from the exams, the intra-group alpha diversity and inter-group beta diversity were analyzed.

From this metric perspective, nine continuous numerical variables were analyzed in each group: the abundance of the Firmicutes and Bacteroidetes phyla in relation to the total phyla present in the feces (PhylABD); the Firmicutes and Bacteroidetes ratio (FiBaRAT); the diversity of bacterial genera (GenDIV); the species richness (SpRIC) between PG and CG and the abundance of each of the following species between PG and CG: *Akkermansia muciniphila* (AM); *Faecalibacterium prausnitzii* (FP); *Bifidobacterium* spp. (BS); *Bacteroides fragilis* (BF) and *Clostridium difficile* (CD).

In the literature, these variables are used to determine the balance of the intestinal bacterial microbiota and represent universal reference values adopted by the Bioma4me* laboratory (Table 1).

### 2.3. Statistical Analysis

The Mann–Whitney and Wilcoxon non-parametric tests (R’s Phyloseq package) (https://joey711.github.io/phyloseq/index.html, accessed on 3 July 2024) were used for the data obtained from the Amplicon sequencing of the groups’ fecal samples. Regarding the variables of the demographic and clinical characteristics, the chi-square test was used for qualitative nominal and Mann–Whitney for quantitative ones.

Six diversity indices were analyzed to determine the alpha diversity (species richness and abundance) of each group: Observed, Chao, Shannon, Simpson, ACE and Fisher [24]. The average relative abundance (ar-Ab) of the species present in the participants’ intestinal bacterial microbiota was also assessed. The difference between the abundance of each group (beta diversity) was assessed using the adjusted *p*-values of the relative frequencies.

**Figure 1:**
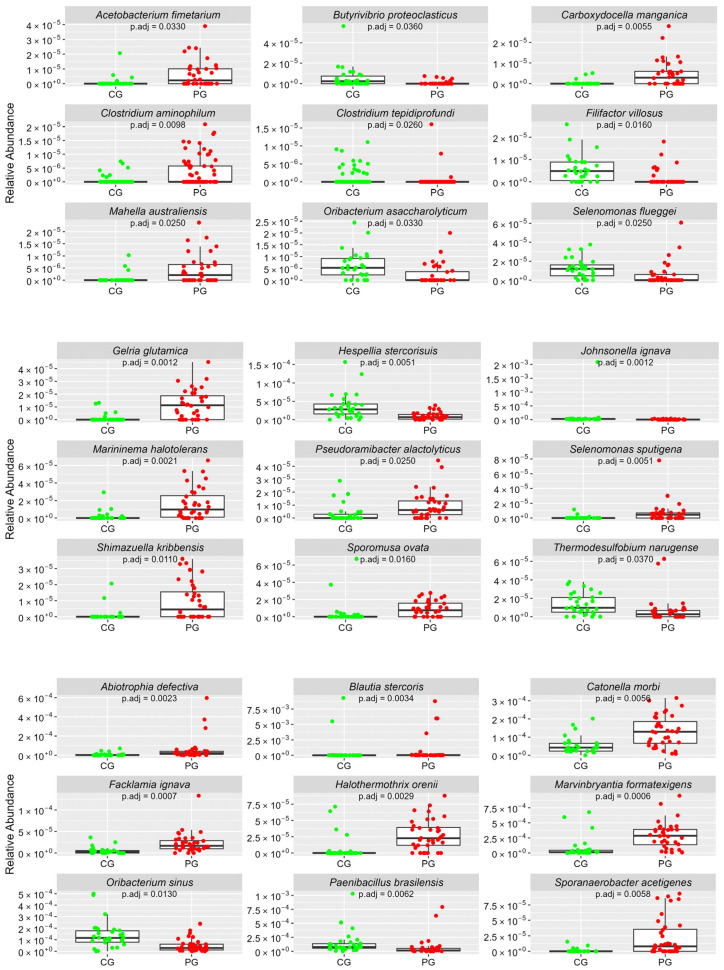
The ar-Ab of bacterial species of the phylum Firmicutes between CG and PG.

To visualize these differences, a compositional circular bar chart was generated with Bray–Curtis ordering (R’s microViz package) (https://www.researchgate.net/figure/Simple-example-of-a-microViz-figure-pairing-an-ordination-plot-of-microbial-samples_fig1_353154579, accessed on 3 July 2024). This graph highlighted the 15 most frequent bacteria with significant differences, allowing for a comparative analysis of the microbiota between PG and CG. 

The *p*-values were adjusted according to the Benjamini–Hochberg procedure [25]. The significance of *p*-values < 0.05 (5% significance level) was considered.

### 2.4. Ethical Aspects

The complete research protocol was approved by the Human Research Ethics Committee of the Santa Casa de Misericórdia de São Paulo, under opinion number 4.816.206. All participants signed the Free and Informed Consent Form before the study began. 

## 3. Results

### 3.1. Sample Description

The demographic and clinical descriptive results were not explored in this study because they were not aligned with the established objectives. However, they present a wealth of material for other researchers seeking correlations between them and the intestinal microbiota sequencing data of the participants, such as the significant differences found in hours of nighttime sleep, amount of vegetable fiber ingested and oscillation between constipation and diarrhea. The *p*-values for the variables gender, age and BMI attest to the similarity between PG and CG (Table 2).

### 3.2. Microbial Composition Analysis

#### 3.2.1. Variables SpRIC and CD

In Relation to the Continuous Variables SpRIC and CD (Table 3), Significant Differences Were Found (*p* < 0.05)

1. Species richness (SpRIC), with PG showing a higher number of taxa compared to CG (*p* = 0.030);
2. Relative abundance of the *Clostridium difficile* species (a pathogenic bacterium related to inflammatory conditions of the large intestine), which is higher in PG (*p* = 0.011).

The ACE, Observed, Chao1 and Fisher alpha diversity index metrics shown in Table 4 confirm the greater microbial diversity in PG, with adjusted *p* < 0.05, that represents the use of Benjamini-Hochberg method that controls the False Discovery Rate (FDR), using sequential modified Bonferroni correction for multiple hypothesis testing. 

#### 3.2.2. Intestinal Bacterial Microbiota Sequencing of the PG and CG Samples

The Statistical Treatment of the Results of the Intestinal Bacterial Microbiota Sequencing of the PG and CG Samples Revealed 52 Species with Significantly Different Average Relative Abundances (ar-Ab) between the Groups (Adjusted *p* < 0.05). The following Distribution of Species by Phylum was Found: Firmicutes (27), Bacteroidetes (7), Proteobacteria (7), Actinobacteria (5), Verrucomicrobia (2), Deferribacteres (1), Acidobacteria (1), Fibrobacteres (1) and Aquificae (1), with 36 Species Being More Abundant in PG and 16 in CG (Table 5).

#### 3.2.3. Significant Difference between CG and PG

**Figure 2:**
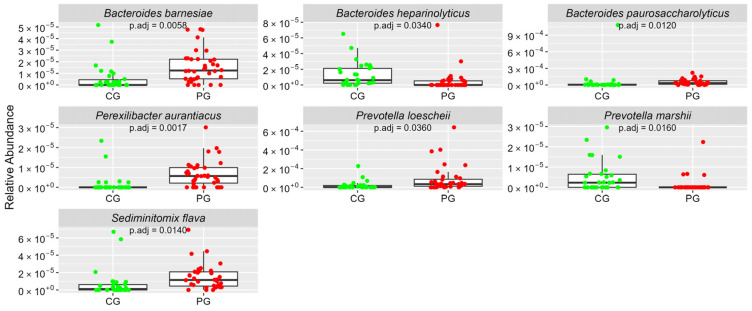
The ar-Ab of bacterial species of the phylum Bacteroidetes between CG and PG.

**Figure 3:**
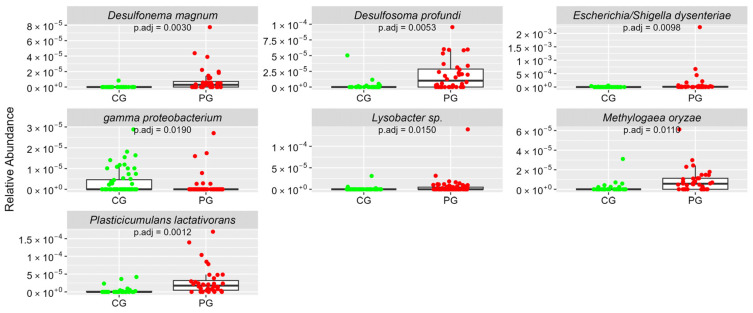
The ar-Ab of the bacterial species of the phylum Proteobacteria between CG and PG.

**Figure 4:**
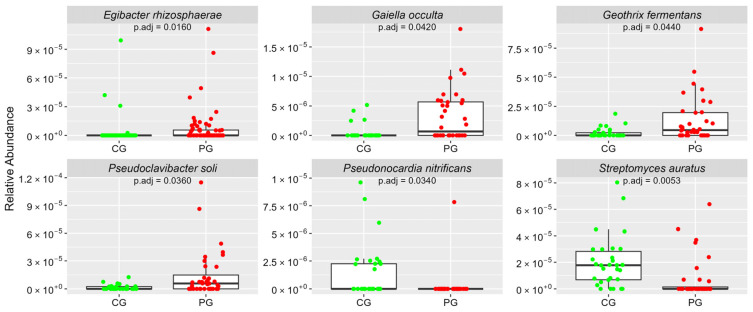
The ar-Ab of the bacterial species of the phylum Actinobacteria between CG and PG.

**Figure 5:**
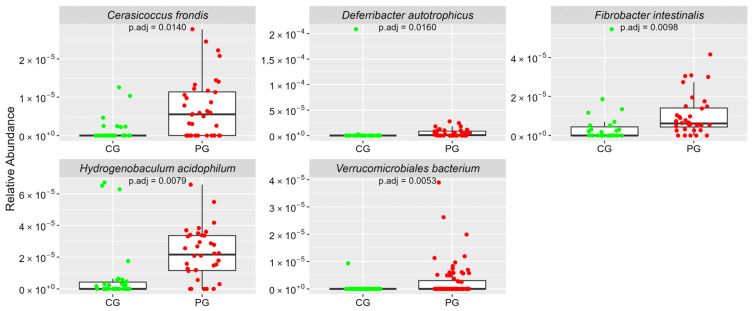
The ar-Ab of bacterial species from other phyla between CG and PG.

Figure 1, Figure 2, Figure 3, Figure 4 and Figure 5 Show the Boxplots of the Average Relative Abundance (ar-Ab) of the Bacterial Species with a Significant Difference between CG and PG (Adjusted *p* < 0.05).

By searching the MEDLINE and SCOPUS health databases, we studied each of the 52 bacterial species with significant differences in ar-Ab between the groups, looking for possible associations with human diseases or health. Intriguingly, we found that six were related to human pathologies and had higher ar-Ab in PG, while three of them were related to health and had higher ar-Ab in CG (see Table 6). 

**Figure 6:**
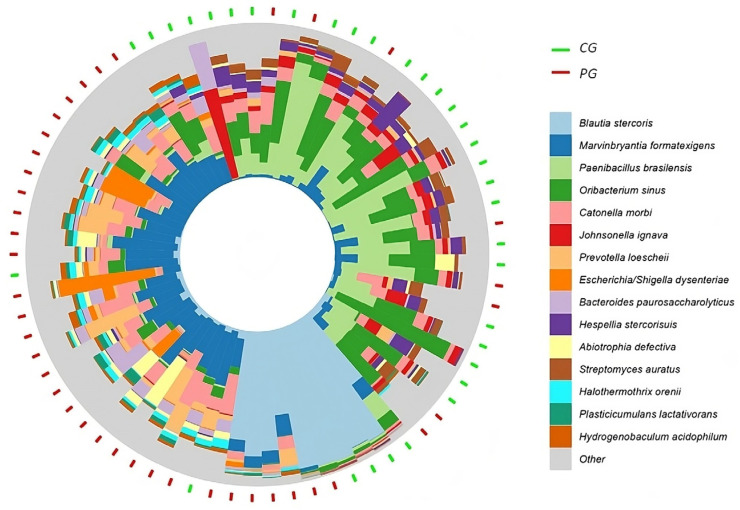
Circular compositional bar chart sorted using the Bray–Curtis sorting angle [35]. This graphic representation of the circle of outer lines shows each participant, with the green rectangles representing CG and the red ones representing PG. In the central projection, the pattern found in the survey is represented according to the color palette, showing 15 more abundant species (6 in CG, 9 in PG) out of the 52 revealed through the diversity indices applied with adjusted *p* differences < 0.05.

After applying the circular compositional bar chart classified using the Bray–Curtis sorting angle [35] in R’s microViz package; https://www.researchgate.net/figure/Simple-example-of-a-microViz-figure-pairing-an-ordination-plot-of-microbial-samples_fig1_353154579, accessed on 3 July 2024, the distribution pattern of CG and PG (Figure 6) is obtained by analyzing the 15 species with the lowest adjusted *p*-values out of the 52 species with significant differences between the groups. A higher ar-Ab of the following species was found in PG: *Blautia stercoris*, *Marvinbryantia formatexigens*, *Catonella morbi*, *Prevotella loescheii*, *Escherichia-Shigella dysenteriae*, *Abiotrophia defectiva*, *Halothermothrix orenii*, *Plasticicumulans lactativorans*, *Bacteroides paurosaccharolyticus*, no CG, *Paenibacillus brasilensis*, *Oribacterium sinus*, *Johnsonella ignava*, *Hespellia stercorisuis*, *Streptomyces auratus*, and *Hydrogenobaculum acidophilum*. 

No significant differences were found between CG and PG in the variables PhylABD, FiBaRAT, GenDIV, AM, FP, BS and BF (*p* > 0.05).

## 4. Discussion

Significant differences were found in some metrics of alpha and beta intestinal bacterial diversity between the groups, which reinforces previous preclinical and clinical studies regarding the importance of the gut lumbar spine axis. 

The species richness (SpRIC) was higher in PG than in CG. This finding indicates that the generic increase in the species alpha diversity is not always the most important marker in human health conditions. In this direction, we highlight the higher average relative abundance of *Clostridium difficile* in relation to inflammatory conditions of the large intestine, which was higher in PG. Furthermore, we found six species related to human pathologies with greater ar-Ab in PG and three related to human health with greater ar-Ab in CG among the 52 species with different values of average relative abundance (ar-Ab). Therefore, these results reinforce the importance of composition, that is, the role of each species, its metabolites and the relationship between them and the host, as the main marker when compared to isolated richness. Furthermore, the lack of data in the literature regarding the 43 species, whose effects on the human body remain poorly understood, points to the importance of more microbiological studies that correlate bacterial species with human physiology.

We could see a distributive pattern from the graphical representation of circular compositional bars classified via the Bray–Curtis ordination angle applied to the 52 species with significant differences in ar-Ab. In summary, the graph demonstrates that the intestinal bacterial composition of people with and without chronic low back pain are different, proving the initial hypothesis of this study that there is an association between the individual intestinal microbiome and the chance of developing low back pain. 

This study has some limitations in its development. The participants did not undergo imaging tests. In addition to the focus of the study being pain, the frequent clinical radiological dissociation established in relation to low back pain methodologically underpins this approach [36]. This dissociation is marked by cases of the absence of vertebral structural alterations in individuals with chronic low back pain [37]; additionally, when the alterations are present, the cases tend to be non-specific or involve unequivocal causes of pain [38]. Furthermore, we uncovered the presence of structural alterations, such as disc degeneration, in 37% of asymptomatic individuals between 20 and 30 years old and in 68% between 40 and 50 years old [39]. This finding includes asymptomatic people with vertebral structural alterations who show no alterations on imaging tests when they develop chronic low back pain. Due to technical operational limitations, sequencing was not carried out using the Shotgun technique, which otherwise would have made it possible to know all the microorganisms present in the sample analyzed and identify them precisely at the strain level. Moreover, the analysis of the elements actually transcribed derived from the microbiome or metatranscriptogenomics was not carried out, which could have elucidated the differential expression of genes in specific situations, thereby expanding knowledge regarding the relationship between the microbiome and the host [40,41]. 

## 5. Conclusions

The research carried out shows a significant difference (*p* < 0.05 and adjusted *p* < 0.05) between PG and CG for some of the taxonomic metrics evaluated and the absence for others. This information could serve as a reference for future similar research aimed at assessing the existence of a possible gut microbiota signature in chronic low back pain. Thus, more studies are needed to confirm possible associative and/or causal correlations and enable a better understanding of the molecular mechanisms that justify it, which could lead to the proposal of more effective and personalized treatments for the leading cause of years lived with disability worldwide.

## Figures and Tables

**Table 1:** Bioma4me* laboratory reference values for selected indicators. AM, FP and BS are associated with health, while BF and CD are associated with disease.

Indicator	Reference
Phylum	
Abundance of Firmicutes and Bacteroidetes in relation to other phyla (PhylABD)	85–95% [15]
Firmicutes and Bacteroidetes Ratio (FiBaRAT)	0.7–1.0 [16]
Gender	
Gender diversity (GenDIV)	Greater than 7.0 [17]
Species	
Species richness (SpRIC)	Greater than 540 [18]
Protective	
*Akkermansia muciniphila* (AM)	1–5% [19]
*Faecalibacterium prausnitzii* (FP)	5–12% [20]
*Bifidobacterium* spp. (BS)	1–6% [21]
Pathogenic	
*Bacteroides fragilis* (BF)	Less than 0.5% [22]
*Clostridium difficilis* (CD)	0 [23]

Bioma4me*: https://biomagenetics.com.br/quem-somos.html, accessed on 3 July 2024.

**Table 2:** Descriptive demographic and clinical data of the sample between the control group (CG) and the low back pain group (PG).

Variables	CG		PG		*p*
Gender: M/F: n, (%)	11/22 (33.3/66.6)		16/24 (40/60)		Chi-squared = 0.1181, df = 1 *p* = 0.7311
Age: mean (SD)	39.79 ± 7.73		38.58 ± 8.1		Mann–Whitney = 720.5, *p* = 0.5055
* Ethnicity: W/B/other, n, (%)	19, 9, 5 (57.5/27.2/15.1)		27, 11, 2 (67.5/27.5/5.0)		Chi-squared = 6.2703, df = 2 *p* = 0.0435
* Average income (SD)	R$ 10,636.70 ± 14,660.44	USD 1970.96 ± 2716.55	R$ 6161.00 ± 6868.45	USD 1141.62 ± 1272.71	Mann–Whitney = 637.5 *p* = 0.2105
* BMI: mean (SD)	23.6 ± 2.5		25.2 ± 2.9		Mann–Whitney = 457 *p* = 0.0248
Hours of sleep at night	<6 h: 9 6–7.5 h: 22 >7: 1		<6 h: 18 6–7.5 h: 14 >7: 5		Chi-squared = 7.1195, df = 2 *p* = 0.0284
*N Meals/day: mean (SD)	3.54 ± 0.9		3.5 ± 1.0		Chi-squared = 7.5132, df = 4 *p* = 0.1111
*N Amount of fiber: R/Po: n, (%)	21, 12 (63.6/36.4)		13, 27 (32.5/67.5)		Chi-squared = 5.0875, df = 1 *p* = 0.0241
*N Liq at mealtimes: Y/N: n, (%)	12, 21 (36.7/63.6)		19, 21 (47.5/52.5)		Chi-squared = 0.19136, df = 1 *p* = 0.6618
*N Larger meal: M/A/E: n, (%)	5, 23, 5 (15.1/69.7/15.1)		6, 27, 7 (15.0/67.5/17.5)		Chi-squared = 0.16572, df = 2 *p* = 0.9205
* Route of birth: V/C/nd: n, (%)	22, 9, 2 (66.6/27.3/6.0)		24, 12, 4 (60.0/30.0/10.0)		Chi-squared = 0.5157, df = 2 *p* = 0.7720
** Bristol stool scale: mode	3		3		***
** Stool frequency: means, DP	1 (0.7)		1 (1.0)		Chi-squared = 0.5157, df = 2 *p* = 0.0308
** D/C oscillation: Y/N: n, (%)	4, 29 (12.1/87.9)		27, 13 (67.5/32.5)		Chi-squared = 20.487, df = 1 *p* = 6.003 × 10^−6^

Legend: M/F male/female; SD standard deviation; W/B white/brown; BMI body mass index; R/Po rich/poor; Liq liquid; Y/N yes/no; M/A/E morning/afternoon/evening; V/C/nd vaginal/cesarean section/no data; n number; D/C diarrhea and constipation. * Information obtained from the research clinical form. *N Information obtained from the clinical research form nutrition survey. ** Information obtained from the stool characteristics form. *** No significant differences; 6.003 × 10^−6^ = 0.000006003.

**Table 3:** Shows the species richness and relative abundance of *Clostridium difficile* in CG and PG.

Variables	CG	PG	*p* < 0.05
SpRIC n (dp)	869.79 (182.520)	962.93 (196.400)	0.030
CD m-ppm (dp)	0.001 (0.002)	0.003 (0.003)	0.011

n: absolute number; m-ppm: mean in parts per million; dp: standard deviation.

**Table 4:** Mean (m) and standard deviation (SD) of patient diversity indices between CG and PG (adjusted *p* < 0.05).

Diversity Index	CG m (dp)	PG m (dp)	Adjusted *p*
ACE	1192.25 (219.06)	1330.47 (252.54)	0.0263
Observed	844.33 (165.66)	951.28 (191.93)	0.0272
Chao1	1188.68 (224.93)	1334.59 (253.37)	0.0263
Fisher	105.31 (24.87)	129.58 (29.48)	0.0045

m: mean; (dp): standard deviation.

**Table 5:** The 52 bacterial species with significant differences (adjusted *p* < 0.05) in terms of average relative abundances (ar-Ab) and mean-standard deviation (sd), with 36 more abundant in PG and 16 in CG.

Taxonomy (Phylum and Species)	CG	PG	Adjusted *p*
**Phylum Firmicutes**	**ar-Ab (sd)**	**ar-Ab (sd)**	
*Blautia stercoris*	4.97 (19.43)	7.11 (20.22)	0.0034
*Paenibacillus brasilensis*	1.46 (2.02)	0.68 (1.65)	0.0062
*Oribacterium sinus*	1.39 (1.20)	0.49 (0.56)	0.0130
*Johnsonella ignava*	0.93 (3.77)	0.08 (0.11)	0.0012
*Marvinbryantia formatexigens*	0.80 (1.71)	2.91 (2.18)	0.0006
*Catonella morbi*	0.54 (0.46)	1.31 (0.85)	0.0056
*Hespellia stercorisuis*	0.36 (0.35)	0.10 (0.10)	0.0051
*Thermodesulfobium narugense*	0.13 (0.11)	0.07 (0.14)	0.0370
*Selenomonas flueggei*	0.12 (0.10)	0.06 (0.12)	0.0250
*Abiotrophia defectiva*	0.08 (0.16)	0.57 (1.18)	0.0023
*Halothermothrix orenii*	0.07 (0.18)	0.28 (0.23)	0.0029
*Filifactor villosus*	0.06 (0.06)	0.02 (0.04)	0.0160
*Butyrivibrio proteoclasticus*	0.06 (0.11)	0.01 (0.02)	0.0360
*Oribacterium asaccharolyticum*	0.06 (0.06)	0.02 (0.04)	0.0330
*Facklamia ignava*	0.05 (0.08)	0.23 (0.23)	0.0007
*Sporomusa ovata*	0.04 (0.14)	0.09 (0.09)	0.0160
*Pseudoramibacter alactolyticus*	0.03 (0.07)	0.09 (0.10)	0.0250
*Marininema halotolerans*	0.02 (0.06)	0.16 (0.18)	0.0021
*Clostridium tepidiprofundi*	0.01 (0.02)	0 (0.02)	0.0260
*Acetobacterium fimetarium*	0.01 (0.04)	0.07 (0.09)	0.0330
*Mahella australiensis*	0.01 (0.02)	0.04 (0.06)	0.0250
*Clostridium aminophilum*	0.01 (0.02)	0.03 (0.05)	0.0098
*Selenomonas sputigena*	0.01 (0.02)	0.07 (0.14)	0.0051
*Gelria glutamica*	0.01 (0.03)	0.12 (0.11)	0.0012
*Sporanaerobacter acetigenes*	0.01 (0.03)	0.22 (0.31)	0.0058
*Shimazuella kribbensis*	0.01 (0.04)	0.10 (0.12)	0.0110
*Carboxydocella manganica*	0 (0.01)	0.05 (0.06)	0.0055
**Phylum Bacteroidetes**	**ar-Ab (sd)**	**ar-Ab (sd)**	
*Bacteroides paurosaccharolyticus*	0.49 (1.97)	0.51 (0.52)	0.0120
*Prevotella loescheii*	0.20 (0.46)	0.85 (1.38)	0.0360
*Bacteroides heparinolyticus*	0.13 (0.15)	0.05 (0.14)	0.0340
*Sediminitomix flava*	0.07 (0.16)	0.15 (0.15)	0.0140
*Bacteroides barnesiae*	0.06 (0.12)	0.16 (0.14)	0.0058
*Prevotella marshii*	0.05 (0.08)	0.01 (0.04)	0.0160
*Perexilibacter aurantiacus*	0.02 (0.05)	0.07 (0.07)	0.0017
**Phylum Proteobacteria**	**ar-Ab (sd)**	**ar-Ab (sd)**	
*Escherichia/Shigella dysenteriae*	0.04 (0.12)	1.14 (3.88)	0.0098
*Plasticicumulans lactativorans*	0.04 (0.11)	0.3 (0.39)	0.0012
*Gamma proteobacterium*	0.03 (0.06)	0.01 (0.04)	0.0190
*Desulfosoma profundi*	0.02 (0.09)	0.19 (0.24)	0.0053
*Methylogaea oryzae*	0.02 (0.06)	0.09 (0.12)	0.0110
*Lysobacter* sp.	0.01 (0.04)	0.05 (0.17)	0.0150
*Desulfonema magnum*	0 (0.02)	0.09 (0.16)	0.0030
**Phylum Actinobacteria**	**ar-Ab (sd)**	**ar-Ab (sd)**	
*Geothrix fermentans*	0.02 (0.04)	0.13 (0.20)	0.0440
*Streptomyces auratus*	0.2 (0.19)	0.07 (0.15)	0.0053
*Egibacter rhizosphaerae*	0.03 (0.14)	0.07 (0.18)	0.0160
*Pseudoclavibacter soli*	0.02 (0.03)	0.15 (0.25)	0.0360
*Pseudonocardia nitrificans*	0.01 (0.02)	0 (0.01)	0.0340
*Gaiella occulta*	0 (0.01)	0.03 (0.04)	0.0420
**Others**	**ar-Ab (sd)**	**ar-Ab (sd)**	
*Hydrogenobaculum acidophilum*	0.08 (0.20)	0.22 (0.16)	0.0079
*Deferribacter autotrophicus*	0.07 (0.38)	0.05 (0.07)	0.0160
*Fibrobacter intestinalis*	0.04 (0.11)	0.10 (0.10)	0.0098
*Cerasicoccus frondis*	0.01 (0.03)	0.07 (0.08)	0.0140
*Verrucomicrobiales bacterium*	0 (0.01)	0.03 (0.06)	0.0053

Average relative abundance (ar-Ab) of bacterial species in parts per million followed by the standard deviation in parentheses (sd), which showed a significant difference between CG and PG (adjusted *p* < 0.05).

**Table 6:** The species with significantly different ar-Ab between CG and PG (adjusted *p* < 0.05) and association with pathology or human health.

Bacterial Species	CG ar-Ab	PG ar-Ab	*p*	Association with Pathology or Health
*Filifactor villosus* *	0.06 (0.06)	0.02 (0.04)	0.0160	Inversely associated with the severity of Alzheimer’s disease [26]
*Gelria glutamica* **	0.01 (0.03)	0.12 (0.11)	0.0012	Glutamate-degrading bacteria [27]
*Johnsonella ignava* *	0.93 (3.77)	0.08 (0.11)	0.0012	Inversely associated with carotid plaques and inflammatory activation [28]
*Pseudoramibacteralactolyticus* **	0.03 (0.07)	0.09 (0.10)	0.0250	Associated with endodontic infections [29]
*Selenomonas sputigena* **	0.01 (0.02)	0.07 (0.14)	0.0051	Associated with periodontitis [30]
*Abiotrophia defectiva* **	0.08 (0.16)	0.57 (1.18)	0.0023	Associated with the pathogenesis of endocarditis [31]
*Catonella morbi* **	0.54 (0.46)	1.31 (0.85)	0.0056	Associated with periodontal disease [32]
*Paenibacillus brasilensis* *	1.46 (2.02)	0.68 (1.65)	0.0062	Produces antimicrobial substance against the pathological species Cryptococcus neoformans [33]
*Escherichia*/*Shigella dysenteriae* **	0.04 (0.12)	1.14 (3.88)	0.0098	Causes inflammation and ulceration of the mucosa of the large intestine [34]

* Species related to human health and with greater ar-Ab in CG. ** Species related to human pathologies and with greater ar-Ab in PG.

## Data Availability

All complete intestinal microbiota sequencing data will be made available to all research participants upon reasonable request.
